# Ecophysiological and Biochemical Responses of *Lessonia spicata* to Solar Eclipse-Induced Light Deprivation

**DOI:** 10.3390/plants14121810

**Published:** 2025-06-12

**Authors:** Paula S. M. Celis-Plá, Camilo E. Navarrete, Andrés Trabal, Pablo A. Castro-Varela, Félix L. Figueroa, Macarena Troncoso, Claudio A. Sáez

**Affiliations:** 1 Laboratory of Aquatic Environmental Research (LACER), HUB-Ambiental UPLA, University of Playa Ancha, Valparaíso 2360004, Chile; camilo.navarrete@upla.cl (C.E.N.); andres.trabal@gmail.com (A.T.); macarena.troncoso@upla.cl (M.T.); 2 Departamento de Ciencias y Geografía, Facultad de Ciencias Naturales y Exactas, University of Playa Ancha, Valparaíso 2360004, Chile; 3 Doctorado Interdisciplinario en Ciencias Ambientales, Facultad de Ciencias Naturales y Exactas, University of Playa Ancha, Valparaíso 2360004, Chile; 4 Escuela de Ciencias Agrarias y Veterinarias, University of Viña del Mar, Viña del Mar 7410005, Chile; 5 Departamento de Botánica, Facultad de Ciencias Naturales y Oceanográficas, University of Concepción, Concepción 3040004, Chile; pabcastro@udec.cl; 6 Institute of Blue Biotechnology and Development (IBYDA), Experimental Center Grice Hutchinson, University of Málaga, 29004 Málaga, Spain; felixlfigueroa@uma.es; 7 Departamento de Ciencias del Mar y Biología Aplicada, Facultad de Ciencias, University of Alicante, 03080 Alicante, Spain

**Keywords:** macroalgae productivity, photo-physiology, photoacclimation, environmental stress, kelps

## Abstract

Light variability is a key environmental stressor influencing the physiology and productivity of marine macroalgae. This study examined the ecophysiological and biochemical responses of *Lessonia spicata* (Ochrophyta) during a natural light deprivation event caused by a solar eclipse. We measured the in vivo chlorophyll *a* (Chl*a*) fluorescence, photoinhibition, and photosynthetic capacity, along with the pigment content, phenolic compound accumulation, and antioxidant capacity, to evaluate short-term photosynthetic adjustments. Dark-adapted conditions during the eclipse peak led to reduced photosynthetic and biochemical activity, while post-eclipse recovery involved the increased accumulation of photosynthetic pigments and photoprotective compounds. Carotenoids showed high antioxidant potential under eclipse exposure, contrasting with declines in chlorophyll content and productivity under pre-eclipse high irradiance. This study provides valuable insights into the rapid acclimation mechanisms of *Lessonia spicata* to transient light stress, highlighting its sensitivity and resilience to sudden shifts in solar irradiance. These findings contribute to the broader field of marine macroalgal photobiology and stress physiology, enhancing our understanding of how intertidal brown algae adapt to dynamic environmental conditions.

## 1. Introduction

During a solar eclipse, the Moon aligns between the Earth and the Sun, partially or completely obstructing sunlight and casting a shadow over the Earth, temporarily blocking solar irradiance. This phenomenon takes place when the Sun, Moon, and Earth are aligned [1], resulting in abrupt changes in the amount of solar radiation reaching the Earth’s surface [2]. Solar radiation—comprising primarily photosynthetically active radiation (PAR, 400–700 nm) and ultraviolet (UV) radiation (UVB, 280–315 nm; UVA, 315–400 nm)—is a critical environmental factor regulating primary productivity in photoautotrophic organisms and maintaining the ecological balance of marine ecosystems. Sudden variations in irradiance can significantly affect photosynthetic rates [3,4,5].

In the South Pacific Ocean, solar irradiance exhibits strong seasonal variation, ranging from approximately 3000 KJ m^−2^ in winter to 9000 KJ m^−2^ in summer [6,7]. While irradiance data are available for this region, there remains limited knowledge about the ecophysiological responses of marine photoautotrophs to abrupt reductions or fluctuations in solar irradiance—especially during extreme natural events like eclipses. According to NASA, the cycle of the eclipses, particularly focusing on the Saros cycle (visibility in South America) is a period of approximately 6585.3 days (18 years, 11 days, and 8 h) (https://science.nasa.gov/eclipses/, accessed 6 June 2025). This is particularly relevant in temperate rocky shore ecosystems in southern Chile, where brown macroalgae are key habitat-forming species and major primary producers [6,8]. These macroalgae are regularly exposed to both daily and seasonal variations in PAR and UV radiation. Although UV radiation in excess is known to be harmful to marine organisms [9], macroalgae display varying degrees of stress tolerance. This tolerance is species-specific and depends on factors such as morphology, shore position (intertidal vs. subtidal), and life cycle stage [9]. Research on brown macroalgae has revealed diverse ecophysiological responses to irradiance stress, including the production of photoprotective compounds. Carotenoids function not only as accessory pigments in light harvesting, but also as highly efficient scavengers of reactive oxygen species (ROS). Indeed, polyphenols can contribute to energy dissipation and exhibit strong antioxidant activity, both of which are essential defense strategies against solar radiation stress in kelp species [6,10].

*L. spicata* is a dominant intertidal kelp and a key bioengineering species of significant ecological and economic importance in central and southern Chile [7,11]. However, this species is regularly subjected to dynamic changes in both PAR and UV radiation, with excessive UV exposure known to be particularly harmful [11,12]. In this way, the polyphenol contents and photosynthetic pigments reported in *L. spicata* increased consistently during spring and summer over a two-year study along the central coast of Chile [6,7]. These findings highlight the central role of photoprotective compounds in enhancing tolerance to elevated solar radiation in brown macroalgae. Such physiological responses are critical for understanding how environmental stressors influence the development, abundance, and spatial distribution of macroalgal species.

To further explore its photophysiological plasticity, we assessed the ecophysiological and oxidative stress responses of *L. spicata* during a unique natural event—a total solar eclipse. We hypothesized that this abrupt, short-term light deprivation would trigger enhanced ecophysiological adjustments, including the elevated production of the compounds of the secondary metabolism as Chla–Chl c–Carotenoids–Poly.

## 2. Results

### 2.1. Environmental Conditions

**Figure 1:**
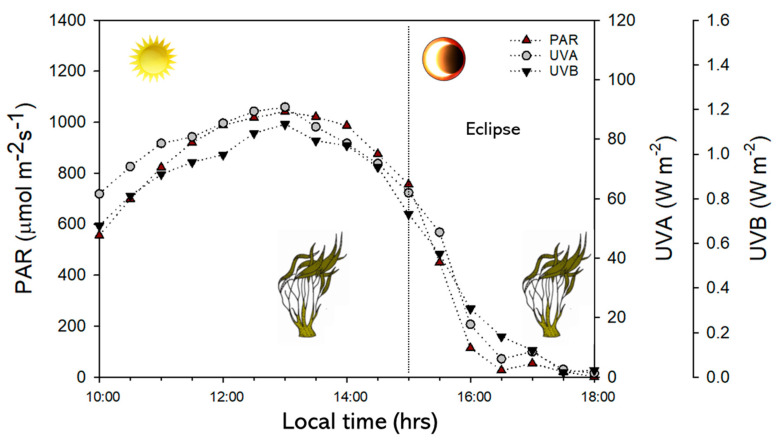
Photosynthetic activate radiation (PAR) and ultraviolet A and B (UVA-UVB) radiation evolution during the experimental period of eclipse phenomenon at Cochoa beach in Valparaíso—Chile.

During the experimental period, the PAR, UVA, and UVB doses were 2380 KJ m^−2^, 745 KJ m^−2^, and 11 KJ m^−2^, respectively (Table 1 and Figure 1). The temperature was 14.5 ± 0.24 °C, the pH values were 7.9 ± 0.9, and the salinity values were 31.95 ± 0.42 PSU (practical salinity unit) (Table 1).

### 2.2. Photosynthetic Performance

**Figure 2:**
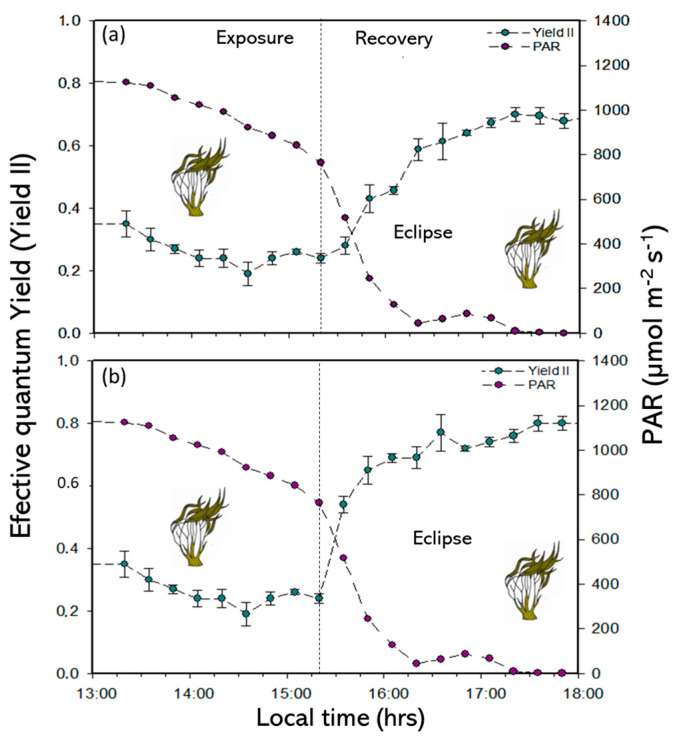
(**a**) Effective quantum yield (Yield II) under non-mesh and (**b**) Yield II under mesh treatments in sun exposure and eclipse phenomenon in *L. spicata*.

**Figure 3:**
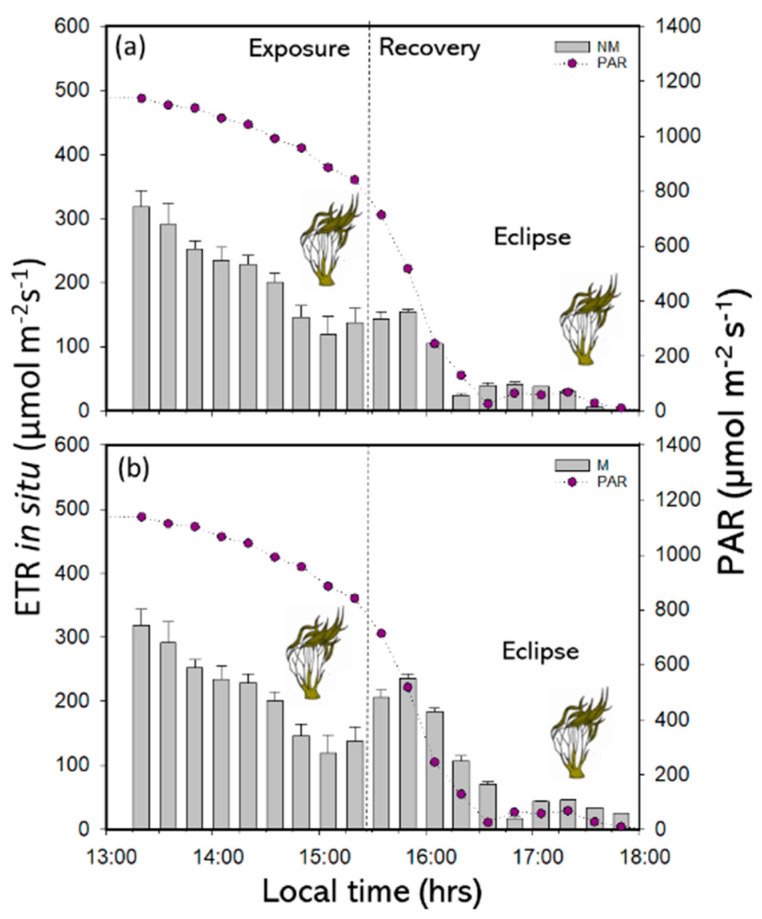
(**a**) In situ electron transport rate (ETR*_in situ_*) with non-mesh treatments and (**b**) ETR*_in situ_* with mesh treatments under sun exposure and eclipse phenomenon in *L. spicata*.

**Figure 4:**
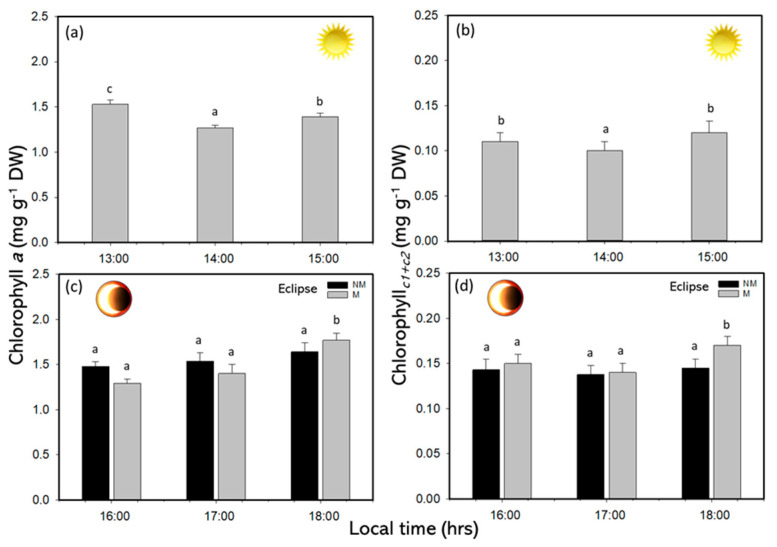
(**a**) Chlorophyll *a* and (**b**) chlorophyll *_c1+c2_* under sun exposure, (**c**) chlorophyll a and (**d**) chlorophyll *_c1+c2_* in recovery phase with non-mesh and mesh treatments in *L. spicata*. Lowercase letters show the significant differences (*p* < 0.05).

The Yield II values in the SE of the experimental period decrease at midday with minimum and maximum values of approximately 0.13 and 0.43, respectively (Figure 2 and Table S1). However, the Yield II values in the RP were higher under the M treatment (approximately 0.79) than under the NM treatment (approximately 0.69) (Figure 3a,b and Table S2). The inhibition and recovery kinetics applied according to the mathematical model. In Yield II, the differences in responses were not significant under SE (Table 2 and Table S1). Similarly, in the RP, P*_fast_* and P*_slow_* had significant differences (*p* < 0.05) for both treatments (M and NM) (Table 2 and Table S2). P*_slow_* and P*_fast_* were higher under the M treatment than under the NM treatment. The *in situ* electron transport rates (ETR*_in situ_*) had significant differences between exposure to the solar eclipse and light treatments (*p* < 0.05). In the SE, the ETR*_in situ_* decreased in the central hours of the day, with a minimum and maximum of 128 and 400 µmol e^−^m^−2^ s^−1^, respectively (Figure 4 and Table S1). In the RP with both M and NM treatments, the ETR*_in situ_* increased at the initial time of phases with 218 nm and 366 µmol e^−^m^−2^ s^−1^-M (Figure 3a,b and Table S2). However, the ETR*_in situ_* decreased abruptly under the exact time of solar exposure at 16:35 h (Local Chilean time) with 20 µmol e^−^m^−2^ s^−1^values (Figure 4 and Table S2).

### 2.3. Biochemical Responses

**Figure 5:**
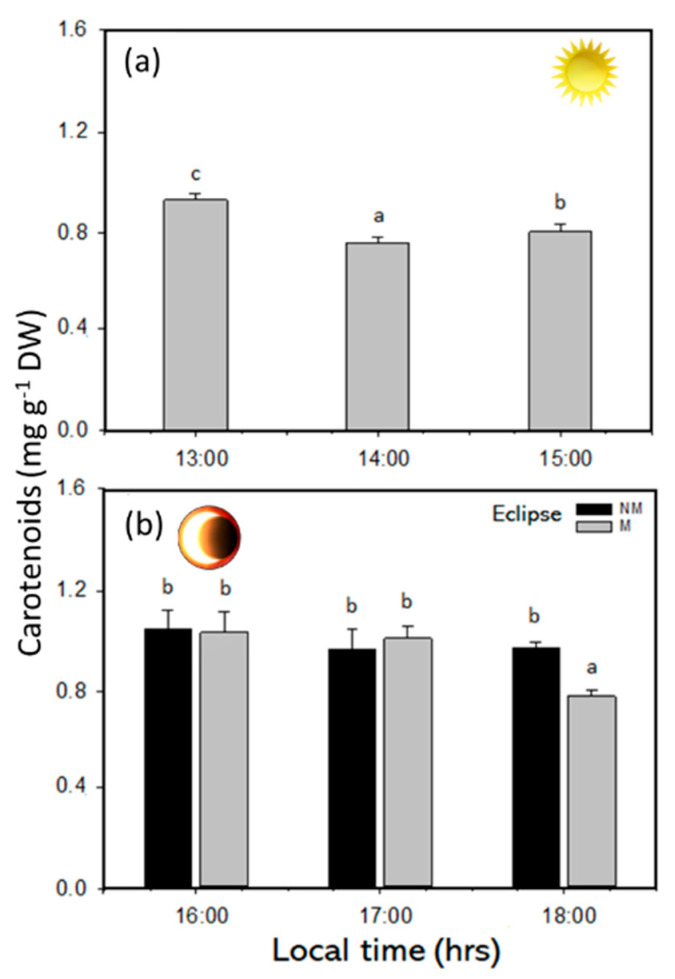
(**a**) Total carotenoids under sun exposure and (**b**) total carotenoids in the recovery phase, with non-mesh and mesh treatments in *L. spicata*. Lowercase letters show significant differences (*p* < 0.05).

Chl*a*, Chl*_c1+c2_*, and carotenoid contents in the SE phase had significant differences during the solar eclipse phenomenon (*p* < 0.05). Both pigment Chl*a* and Chl*_c1+c2_* decreased at midday with higher solar irradiance (Figure 4a,b). Additionally, at the end of the experiment period in NM and M treatment, the pigments contents were higher (Figure 4c,d and Tables S3 and S4). The TCs in the SE phase presented a significant decrease during the first hours of the experimental period, with concentrations of 1.03 mg g^−1^ DW at 13:00 h and values of approximately 0.92 mg g^−1^ DW at 15:00 h (Figure 5a and Table S3). During the RP, significant differences were recorded at the end of the experiment, with values of approximately 0.88 mg g^−1^ DW for the M treatment and 1.05 mg g^−1^ DW for the NM treatment (Figure 5b and Table S4). Additionally, a positive correlation (r = 0.588) was observed in total carotenoids with H_2_O_2_ (Table S5).

**Figure 6:**
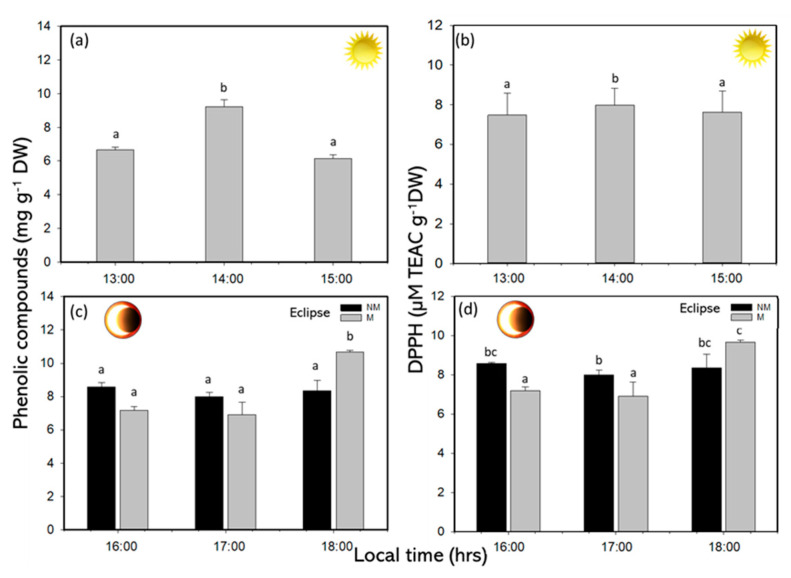
(**a**) Phenolic compounds and (**b**) antioxidant capacity (DPPH) under sun exposure, (**c**) phenolics compounds and (**d**) antioxidant capacity in recovery phase or eclipse phenomenon, under non-mesh and mesh treatments in *L. spicata*. Lowercase letters show the significant differences (*p* < 0.05).

The concentrations of PCs showed significant differences under SE, with higher values of approximately 9.22 mg g^−1^ DW at midday (Figure 6a and Table S3). Indeed, under a solar eclipse in the RP, the PC content was significantly higher, at approximately 8.36–10.66 mg g^−1^ DW in NM compared to M treatment, respectively (Figure 6c and Table S4). The DPPH was significantly higher, at approximately 67% in the central time (Figure 6b and Table S3), and in the RP, the DPPH was significant at the end the experimental time in M treatments (Figure 6d and Table S4).

**Figure 7:**
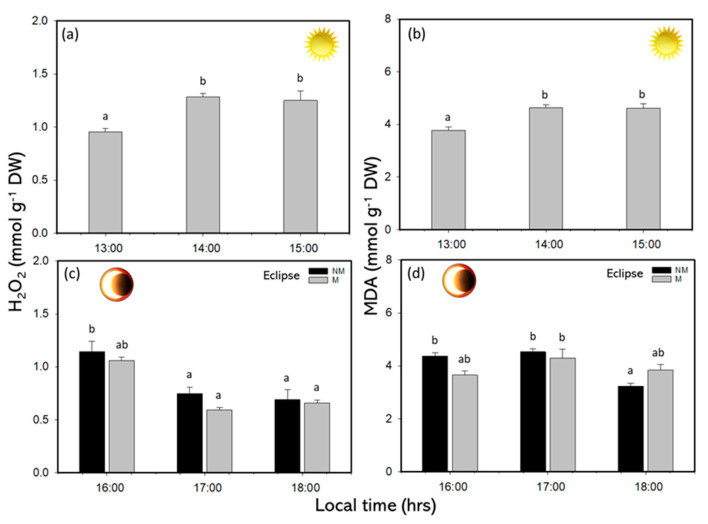
(**a**) Concentration of hydrogen peroxide (H_2_O_2_) and (**b**) malondialdehyde (MDA) during sun exposure, (**c**) concentration of hydrogen peroxide (H_2_O_2_) and (**d**) malondialdehyde (MDA) in recovery phase or eclipse phenomenon in *L. spicata*, according to non-mesh and mesh treatments. Lowercase letters show the significant differences (*p* < 0.05).

**Figure 8:**
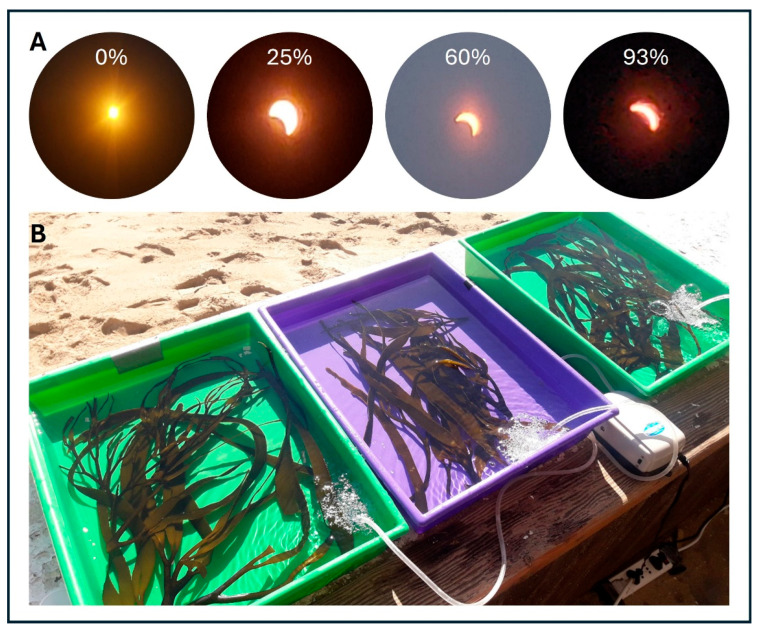
(**A**) Eclipse phenomenon evolution in July of 2019, with different percentage evolution of coverage (0, 25, 60, and 93%) and (**B**) experimental design with *Lessonia spicata*.

The H_2_O_2_ concentrations significantly increased during SE at midday, with values of approximately 1.25–1.28 mmol g^−1^ DW (Figure 7a and Table S3). Indeed, in the RP, the H_2_O_2_ concentrations decreased significantly, with values of approximately 0.5 mmol g^−1^ DW at the end of the experiment in both treatments (M and NM) (Figure 7c and Table S4). The MDA concentration significantly increased during SE at midday, with concentrations between 3.77 and 3.83 mmol g^−1^ DW (Figure 8B and Table S3). Under eclipse, the MDA was significantly higher in M and NM treatments in the middle time, with approximately 4.37 mmol g^−1^ DW (Figure 7d and Table S4).

## 3. Discussion

Few studies have addressed the ecophysiological and biochemical responses of seaweeds to solar eclipses, even though fluctuations in solar radiation are primary drivers of photosynthesis and key determinants of the carbon balance in photoautotrophic organisms. A solar eclipse represents a unique natural experiment, characterized by rapid and transient alterations in light and temperature, which can significantly influence photosynthetic regulation. However, the rarity and brief duration of total solar eclipses pose logistical challenges for experimental evaluation, and thus, such effects remain largely unexplored [14]. Here, we evaluated the ecophysiological responses of *Lessonia spicata* to the total solar eclipse in Valparaíso Bay. Notably, we observed an increase in photosynthetic activity and a marked recovery following the eclipse event. Comparable findings have been reported in vascular plants. For instance, Sambandan et al. [5] observed decreased chlorophyll content and increased carotenoid levels in *Portulaca oleracea* during the solar eclipse. Similarly, Beverly et al. [15] reported suppressed photosynthetic activity in *Artemisia tridentata* during the total solar eclipse in the United States.

In general, macroalgae are frequently exposed to environmental stressors and have acquired diverse acclimation mechanisms. These include ecophysiological responses that mitigate damage and maintain metabolic function under fluctuating irradiance and other stress conditions [16,17]. In this study, the ecophysiological parameters’ effective quantum yield (Yield II) and electron transport rate measured during solar exposure were 0.22 ± 0.04 and 232.8 ± 41.7 μmol m^−2^ s^−1^, respectively, at midday under full solar irradiance. Comparable values have been reported in other brown macroalgae such as *Macrocystis pyrifera* [4,18] and *Laminaria saccharina* [19], where increased solar irradiance led to reduced photosynthetic performance—indicative of photoinhibition. This downregulation is widely recognized as a photoprotective mechanism in intertidal algae exposed to highlight stress [6,17].

Following the eclipse event, a significant recovery in photosynthetic activity was observed, with Yield II increasing to values between 0.69 and 0.78. These recovery levels are consistent with previous studies. For example, [16] reported post-stress Yield II values of around 0.61 in *Petalonia fascia*, and Celis-Plá et al. [20] documented increases of up to 0.75 in *Ericaria selaginoides* (ex*-Cystoseira tamariscifolia*) within low-exposure tidal pools (~50 cm depth). In contrast, specimens from exposed rocky shores displayed slightly lower values (~0.72), emphasizing the influence of the microhabitat on photophysiological recovery. Under solar eclipse conditions, the ETR*_in situ_* decreased slightly, with values of 153.7 ± 1.1 μmol e^−^ m^−2^ s^−1^ in the M treatment, and 227.6 ± 1.4 μmol m^−2^ s^−1^ in the NM treatment. Celis-Plá et al. [20] showed values in *E. selaginoides* of 200–300 μmol m^−2^ s^−1^ in winter and 300–400 μmol e^−^ m^−2^ s^−1^ in summer. Rapid- and slow-phase response ratios were also assessed. Under full irradiance, the P*_slow_* and P*_fast_* values were approximately 0.6 and 2.4, respectively. These values align with those reported by Hanelt et al. [13] for *L. saccharina*, which exhibited P*_fast_* = 0.48 and P*_slow_* = 0.22, suggesting the presence of photoinhibition as a photoprotective response in intertidal brown algae [21]. In this study, under eclipse conditions, P*_fast_* differed significantly between treatments: 0.62 in NM versus 1.19 in M. Similar distinctions in recovery kinetics were observed by Hanelt et al. [13], who reported P*_fast_* values of 0.3 and 0.2 in algae from shallow and subtidal waters, respectively. These observations support the interpretation that intertidal macroalgae exhibit rapid photoacclimation and efficient recovery mechanisms under fluctuating irradiance. Such dynamics are also supported by findings in *Ulva rotundata*, where subtidal versus intertidal forms showed contrasting PSII photoinhibition responses under light stress [22,23].

The pigment content in *Lessonia spicata* decreased under full solar irradiance, with values of approximately 1.5 mg g^−1^ DW for Chl*a*, 0.10 mg g^−1^ DW for Chl*c_*1*+_c_*2*_*, and 0.88 mg g^−1^ DW for the TCs. Similar trends were observed by Celis-Plá et al. [6] during a diel cycle experiment, where the pigment levels declined during midday hours in summer. This reduction represents a photoacclimation mechanism in macroalgae, as short-term adjustments in the pigment concentration serve to modulate light absorption and minimize photodamage. During the solar eclipse event, the pigment levels increased, reaching 1.77 mg g^−1^ DW (Chl*a*), 0.17 mg g^−1^ DW (Chl*c_*1*+_c_*2*_*), and 1.00 mg g^−1^ DW (carotenoids). These results suggest that *L. spicata* enhances pigment accumulation during periods of reduced irradiance to optimize light capture. Short-term fluctuations in the photosynthetic pigment content, as well as in mycosporine-like amino acids (MAAs), have been previously documented in several macroalgal species in response to changing light conditions [24,25,26,27]. Borum et al. [28] found that *Laminaria saccharina* increased the Chl*a* concentration under reduced solar radiation, supporting the idea that pigment upregulation is a compensatory mechanism to enhance light harvesting. The observed increase in carotenoids during the eclipse further supports their role in photoprotection, particularly in reactive oxygen species (ROS) scavenging [29]. In this way, Roach et al. [30] demonstrated in freshwater algae that the diurnal cycling of hydrogen peroxide (H_2_O_2_) was positively correlated with xanthophyll levels. Because thylakoid membranes contain a greater amount of xanthophylls than can be bound by light-harvesting complex proteins, this implies the existence of a free pool of xanthophylls dedicated to abiotic stress protection [31]. Similarly, Karkhaneh et al. [32] reported that the highest levels of fucoxanthin in species such as *Dictyota indica*, *Padina tenuis*, *Colpomenia sinuosa*, and *Lyengaria stellata* occurred during winter, which was attributed to reduced solar irradiance and an enhanced demand for photoprotection.

The polyphenols in *Lessonia spicata* increased significantly during peak solar exposure, reaching values of 9.2 mg g^−1^ DW at midday. During the solar eclipse phase, the PC values ranged between 8.4 and 10.7 mg g^−1^ DW, indicating a consistent elevation of photoprotective compounds under changing irradiance conditions. Similar responses have been previously reported in brown macroalgae, where increased PC levels serve as a photoprotective strategy under high solar radiation, particularly in Mediterranean ecosystems such as the southern Iberian Peninsula [8,10]. Daily fluctuations in the PC content have also been documented in species like *Macrocystis integrifolia*, *E*. *selaginoides*, and *Macrocystis pyrifera* [6,10,33]. The antioxidant capacity was highest at the end of the solar eclipse phase, showing a positive correlation with the polyphenol concentration. This correlation aligns with findings by Celis-Plá et al. [6] and Figueroa et al. [8] emphasizing the role of polyphenols in mitigating oxidative stress. Reactive oxygen species (ROS) concentrations increased proportionally with solar irradiance, reaching approximately 1.3 mmol g^−1^ DW at midday, but declined to around 0.5 mmol g^−1^ DW during the eclipse. Although ROS are naturally produced as a by-product of aerobic metabolism, elevated levels are indicative of oxidative stress induced by intense solar radiation [34]. These findings suggest that stress conditions were alleviated during the eclipse. Malondialdehyde (MDA) concentrations, an indicator of lipid peroxidation and membrane damage, were approximately 4–5 mmol g^−1^ DW under full solar exposure, and decreased to 3–4 mmol g^−1^ DW by the end of the experiment. Shiu et al. [35] reported similar responses in *Ulva fasciata*, where membrane damage was associated with UVB exposure exceeding 1.8 W m^−2^ for prolonged periods. In contrast, the values recorded in this study were lower and not sufficient to induce cellular damage, likely due to the temporary reduction in irradiance during the solar eclipse and the concomitant increase in photoprotective compounds in both mesh and non-mesh treatments.

Overall, these results suggest that solar radiation during the eclipse did not represent a critical oxidative stress factor, since as the light intensity decreases, the efficiency of the light reaction decreases, and the algae can adjust their photosynthetic mechanisms to adapt to this change, such as adjusting their photoprotection systems against free radicals. It was observed that the activity of non-enzymatic antioxidant systems, specifically carotenoids and polyphenols, was enhanced, which contributed to the reduction in ROS such as hydrogen peroxide. In this regard, it is possible to suggest that the stress state is not necessarily conditioned by high light; it is also possible due to a deficiency in the temperature or nutrients where the electron flow is unbalanced, and electron leaks towards molecular oxygen (O_2_) can occur [36,37]. Similar protective responses have been observed in *Ecklonia cava* [36] and *Sargassum hystrix* [37], highlighting the efficiency of photoprotective mechanisms in brown macroalgae under fluctuating light regimes.

## 4. Methods and Materials

### 4.1. Sampling and Experimental Design

In the intertidal zone of Playa Cochoa, Valparaíso Bay (32°57′19.0″ S; 71°32′52.4″ W), specimens of *Lessonia spicata* (Suhr) Santelices (Ochrophyta) were collected at 0.1 to 0.4 m above sea level, with a minimum separation of 2 m between individuals. The samples were collected in the morning (08:00 h), coinciding with a total solar eclipse, covering approximately 95% of the sampling site on 2 July 2019. During the experiment period, six thalli of *L. spicata* (each ~10 g fresh weight) were individually placed in 2.5 L plastic containers filled with filtered seawater. The temperature of the containers was controlled by aeration to simulate natural intertidal conditions (Figure 8). Given the rarity and precise timing of such events, the experimental time points were not randomly selected but were strategically chosen to coincide with the eclipse phases (pre-eclipse, peak obscuration, and post-eclipse) to assess the short-term physiological and biochemical responses of *Lessonia spicata* under natural light deprivation conditions. Indeed, the experiment’s design consisted of two main phases: (i) solar exposure (SE), where all six replicates were exposed to natural solar irradiance between 10:00 and 15:00 local time; and (ii) the recovery phase (RP), from 15:00 to 16:00, coinciding with the peak and post-peak of the solar eclipse. During the RP, three replicates were subjected to attenuated solar irradiance (<33%) using mesh screens (mesh treatment—M), while the remaining three replicates were placed under complete solar obscuration conditions (non-mesh treatment—NM) (Figure 8). To measure the physiological responses and biochemical assays, all samples were preserved in liquid nitrogen and transported to a laboratory at HUB Ambiental—Playa Ancha University.

### 4.2. Abiotic Parameters

Several environmental parameters were carefully controlled or monitored throughout the experiment to account for dynamic environmental variability. Indeed, the environmental conditions were recorded throughout the day, as described by Quintano et al. [38]. Apogee sensors (Apogee Instruments Inc., Logan, UT, USA) were used to quantify the changes in the spectral composition of solar radiation (PAR, UVA, and UVB). The seawater temperature was recorded using a HOBO Pendant^®^ data logger (Onset Computer Corporation, Bourne, MA, USA). The abiotic parameters (salinity, pH, and conductivity) were measured with a multiparameter instrument (HI 98194, Hanna Instruments, Woonsocket, RI, USA).

### 4.3. Physiological Responses

To evaluate the photosynthetic performance, two fluorimeters were used: (1) MINI_PAM II and (2) JUNIOR_PAM (Walz, Effeltrich, Germany), and the in vivo fluorescence of chlorophyll *a* (Chl*a*) was quantified through the effective quantum yield (Y II), where YII = *Fm’* − *Fo/Fm’* and the basal (*Fo*) and maximum (*Fm’*) fluorescence were measured under light conditions. The *_in situ_* electron transport rate (ETR*_in situ_*) was determined as follows (Equation (1)):

(1) $${ETR}_{\mathrm{in}\;\mathrm{situ}}\;(µmol\;electrons\;m^{-2}\;s^{-1})=YII\times E_{\mathrm{PAR}}\times A\times FII$$

where E*_PAR_* corresponds to the irradiance in each light pulse (µmol m^−2^ s^−1^) and A is the absorptance (relative units), which is the fraction of light absorbed through of the algae thalli and calculated using A = 1 − (*Ef*/*Et*), where *E*_F_ is the irradiance transmitted through the algae thalli and *E*_T_ is the total irradiance, measured with a cosine-corrected PAR sensor (LI-COR Company, Nebraska). F*_II_* is the portion of Chl*a* (0.8) absorbed by brown macroalgae [27].

### 4.4. Mathematical Model

During SE and the RP, the values obtained from the YII were used in the mathematical model according to Hanelt et al. [13]. Therefore, two stages were distinguished in each phase of the model: (1) inhibition (Equation (2)) and (2) recovery (Equation (3)).

(2) Inhibition Y(inh)=Pfast * e^(−kfast * t)+Pslow * e^(−kslow  * t)

(3) Recovery Y(rec)=Fv/Fm−(Pfast * e^(−kfast * t)+Pslow * e^(−kslow * t))

### 4.5. Biochemical Responses

The biomass was washed with filtered and distilled water to remove sand particles, epiphytes, and other undesirable materials. The biomass samples were carefully milled with a grinder machine (MARCA, MODELO). Pigment quantification (Chl*a*, Chl*_c1+c2_*, and total carotenoids: TCs) was performed using a microplate spectrophotometer (SPECTROstar Nano, BMG Labtech, Offenburg, Germany). To evaluate the Chl*a* and Chl*c*, the performance protocols according to [39] were used. The TCs were measured according to Parson et al. [40]. The ratio to convert the mg g^−1^ fresh weight to dry weight (DW) was 2.84 for *L. spicata*.

(4) $$Chla=11.47\times (A_{664}-A_{750})-0.45\times (A_{630}-A_{750})$$

(5) $$Chlc1+c2=22.679\times (A_{630}-A_{750})-3.404\times (A_{664}-A_{750})$$

(6) $$TC=10\times (A_{480}-A_{750})$$

Phenolic compounds (PCs) were extracted from *L. spicata* in 0.25 g FW with 80% methanol and incubated under overnight shaking conditions according to Celis-Plá et al. [21]. Folin–Ciocalteu reagent (Merck KGaA, Darmstadt, Germany) and phloroglucinol (Sigma-Aldrich, Darmstadt, Germany) were added as a standard to measure absorbance at 760 nm with a spectrophotometer and expressed as mg g^−1^ DW.

The antioxidant activity was determined using the 2,2-diphenyl-1-picrylhydrazyl DPPH method [21]. The reference standard used corresponds to the Trolox compound (6-hydroxy-2,5,7,8-tetramethylchroman-2-carboxylic acid, 0–50 μM) [41].

For H_2_O_2_ quantification, 100 mg of a liquid nitrogen-ground *L. spicata* sample was used, and 100 µL of 10% trichloroacetic acid (TCA), 100 µL of 10 mM potassium phosphate buffer (pH 7.0), 100 µL of lysis buffer (Favorgen, Vienna, Austria), and 500 µL of 1 M potassium iodide (KI) were added [4]. Controls were performed with 500 µL of H_2_O instead of 500 µL of KI; the 30% H_2_O_2_ standard (Merck) in the standard curve [0–20 µg mL^−1^].

Lipid peroxidation–Thiobarbituric acid (TBARS) in *L. spicata* were quantified through malondialdehyde (MDA) [4]. Controls were performed with 200 µL of 10% TBA. The 1,1,3,3-tetramethoxypropane standard was used in the standard curve [0–25 µM].

### 4.6. Statistical Analysis

The ecophysiological responses in *L. spicata* were evaluated through one-way ANOVA, with 3 levels: (1) 13:00, (2) 14:00, and (3) 15:00 h [42]. In the RP under darkness, a two-way ANOVA was used with 2 factors—(1) time (13:00–17:00–18:00 h) and (2) light treatments (NM-M). Student–Newman–Keuls tests were performed to assess significant interactions in the ANOVA. The homogeneity of variance was assessed using Cochran’s tests [42]. Pearson’s coefficient was calculated to determine the correlation pattern, and all analyses were performed using RStudio 4.4.3 (R Core Team, 2025, Vienna, Austria).

## 5. Conclusions

In vivo chlorophyll *a* fluorescence proved to be a highly sensitive and effective technique for detecting short-term variations in photosynthetic activity in response to the transient decrease in solar irradiance caused by solar eclipse. The rapid adjustments observed in photosynthetic performance, along with the dynamic accumulation of bioactive compounds—such as photosynthetic pigments and antioxidant metabolites—demonstrate the high acclimation capacity of kelp forests. These findings underscore the physiological plasticity of brown macroalgae in adapting to abrupt fluctuations in light availability and offer novel insights into their ecophysiological resilience. This study provides a valuable foundation for understanding macroalgal responses to rare but ecologically significant phenomena like solar eclipses, and more broadly, to rapid environmental changes. The observed modulation of photosynthetic and protective mechanisms highlights the critical role of these species in maintaining coastal ecosystem functioning amid increasing climate variability. Furthermore, these results have important implications for the large-scale monitoring of macroalgal health in marine ecosystems. The fast and measurable physiological and biochemical responses of *L. spicata* to transient light deprivation events suggest that tools such as in vivo chlorophyll *a* fluorescence and bioactive compound profiling could serve as sensitive, early-warning indicators of environmental stress. Integrating these methods into long-term monitoring frameworks would improve the capacity to track ecosystem responses to stressors such as light variability, heatwaves, or ultraviolet radiation anomalies. Additionally, a deeper understanding of species-specific photoacclimation strategies can enhance predictive models of primary productivity and resilience in coastal systems, contributing to informed conservation, ecosystem restoration, and the sustainable management of marine algal resources.

## Figures and Tables

**Table 1:** Results of measurements of abiotic variables, temperature, pH, salinity, and solar radiation on the day of the experiment with the solar eclipse.

Variables	Values
PAR (KJ m^−2^)	2380.1 ± 10.8
UVA (KJ m^−2^)	745.2 ± 1.3
UVB (KJ m^−2^)	11.3 ± 0.5
Temperature (°C)	12.3 ± 0.1
pH	7.9 ± 0.1
Salinity (PSU)	31.9 ± 0.4

**Table 2:** Results of the application of the [13] model, analysis of the physiological responses in *Lessonia spicata*, comparing the exposure and recovery phases of the experiment in the presence of a solar eclipse. Small letters show significant differences (*p* < 0.05). Asterisks show the significant differences (*p* < 0.05).

	P*_fast_*	K*_fast_*	P*_slow_*	K*_slow_*
Exposure	0.30 ± 0.06	0.01 ± 0.00	0.15 ± 0.07	0.01 ± 0.00
Non-mesh	0.63 ± 0.11 ^a,^*	0.02 ± 0.00	2.04 ± 0.13 *	0.02 ± 0.00
Mesh	1.19 ± 0.10 ^b,^*	0.02 ± 0.00	2.16 ± 0.03 *	0.02 ± 0.00

## Data Availability

Data will be made available on request.

## Supplementary Materials

The following supporting information can be downloaded at: https://www.mdpi.com/article/10.3390/plants14121810/s1. Table S1. ANOVA results in physiological analysis of *Lessonia spicata* under solar irradiance exposure. *p* < 0.05 (**); Table S2. ANOVA results for physiological analysis of *Lessonia spicata* during the solar eclipse, with Mesh and Non-Mesh treatments. *p* < 0.05 (**); Table S3. ANOVA results for biochemical analysis of *Lessonia spicata* under solar irradiance exposure. *p* < 0.05 (**); Table S4. ANOVA results in biochemical analysis of *Lessonia spicata* under solar eclipse with Mesh and Non-Mesh treatments. *p* < 0.05 (**); Table S5. Pearson coefficient (r) between the different variables analyzed under solar eclipse with Mesh and Non-Mesh treatments in *L. spicata*.
