# Supplementary material for: Ecophysiological and Biochemical Responses of Lessonia spicata to Solar Eclipse-Induced Light Deprivation
Source: Plants (Basel). 2025 Jun 12;14(12):1810. doi: 10.3390/plants14121810 (PMC12196580; doi:10.3390/plants14121810)
Supplement: Supplementary file 1 [file plants-14-01810-s001.zip › plants-3674731-supplementary.pdf]

Supplementary Materials

Table S1. ANOVA results in physiological analysis of *Lessonia spicata* under solar irradiance exposure. $p < 0.05$ (**).

		Lessonia spicata Exposure			
		df	*MS*	*F*	*P*
**Y(II)**	*Time*	2	0.011	0.890	0.431
	Res	15	0.012		
**$ETR_{in\ situ}$**	*Time*	2	23009	1.725	0.211
	Res	15	13333		
**P_{fast}**	*Time*	1	0.003	0.127	0.739
	Res	4	0.028		
**K_{fast}**	*Time*	1	0.000	0.340	0.593
	Res	4	0.000		
**P_{slow}**	*Time*	1	0.006	0.205	0.673
	Res	4	0.032		
**K_{slow}**	*Time*	1	0.000	3.516	0.134
	Res	4	0.000		

Res: Residua.l

Table S2. ANOVA results for physiological analysis of *Lessonia spicata* during the solar eclipse, with Mesh and Non-Mesh treatments. $p < 0.05$ (**).

**Y(II)**	*Time (T)*	2	0.041	88.58	**
	treatment (t)	1	0.077	164.82	**
	*T*t*	2	0.004	10.52	**
	Res	12	0.000		
**$ETR_{in\ situ}$**	*Time (T)*	2	44375.0	19939.53	**
	treatment (t)	11	3652.5	1641.22	**
	*T*t*	2	2324.3	1044.39	**
	Res	12	2.2		
**P_{fast}**	*treatment*	1	0.901	9.451	**
	Res	4	0.095		
**K_{fast}**	*treatment*	1	0.000	2.799	0.169
	Res	4	0.000		
**P_{slow}**	*treatment*	1	0.000	0.001	0.969
	Res	4	0.043		
**K_{slow}**	*treatment*	1	0.000	2.674	0.177
	Res	4	0.000		

Res: Residual.

Table S3. ANOVA results for biochemical analysis of *Lessonia spicata* under solar irradiance exposure. $p < 0.05$ (**).

		Lessonia spicata Exposure			
		df	*MS*	*F*	*P*
**Chlorophyll *a***	*Time*	2	**0.049**	**35.0**	**
	Res	6	0.003		
**Chlorophyll *c***	*Time*	2	**0.125**	**2.619**	**
	Res	6	0.001		
**Carotenoids**	*Time*	2	**0.019**	**370.1**	**
	Res	6	0.054		

**Phenolic compounds**	*Time*	2	**1.304**	**0.359**	**
	Res	6	0.362		
**[H_2O_2]**	*Time*	2	**1.242**	**1.392**	**
	Res	6	0.892		
**DPPH**	*Time*	2	**0.013**	**1.396**	**
	Res	6	0.009		
**TBARS**	*Time*	2	**6.557**	**4.064**	**
	Res	6	1.613		

Res: Residual.

Table S4. ANOVA results in biochemical analysis of *Lessonia spicata* under solar eclipse with Mesh and Non-Mesh treatments. $p < 0.05$ (**).

		Lessonia spicata			
		df	***MS***	***F***	***P***
**Chlorophyll *a***	*Time (T)*	2	**0.160**	**7.460**	**
	treatment (t)	1	0.019	0.906	
	*T*t*	2	0.043	2.009	
	Res	12	0.021		
**Chlorophyll *c***	*Time (T)*	2	0.005	1.070	**
	treatment (t)	1	0.010	1.802	
	*T*t*	2	0.036	0.649	
	Res	12	0.005		
**Carotenoids**	*Time (T)*	2	0.041	3.587	**
	treatment (t)	1	0.010	0.928	
	*T*t*	2	0.016	1.438	
	Res	12	0.011		
**Phenolic compounds**	*Time (T)*	2	7.107	12.262	**
	treatment (t)	1	0.017	0.029	
	*T*t*	2	6.325	10.912	**
	Res	12	0.580		
**[H_2O_2]**	*Time (T)*	2	0.181	9.149	**
	treatment (t)	1	0.136	6.897	**
	*T*t*	2	0.013	0.013	
	Res	12	0.019		
**DPPH**	*Time (T)*	2	0.156	17.044	**
	treatment (t)	1	0.017	1.955	
	*T*t*	2	0.071	7.765	**
	Res	12	0.009		
**TBARS**	*Time (T)*	2	12.762	0.921	**
	treatment (t)	1	8.279	1.195	
	*T*t*	2	65.207	4.706	**
	Res	12	6.127		

Res: Residual.

Table S5. Pearson coefficient (r) between the different variables analyzed under solar eclipse with Mesh and Non-Mesh treatments in *L. spicata.*

**Correlations**								
		**DPPH**	**Chlc**	**Chla**	**Carotenoids**	**TBARS**	**H2O2**	**Phenols**
	Pearson Correlation	1	-.189	-.177	.418	-.189	.415	-.425
DPPH	Sig. (2-tailed)		.454	.482	.084	.452	.087	.079
	N	18	18	18	18	18	18	18

Chlc	Pearson Correlation	-.189	1	.571*	-.048	-.194	-.188	.376
	Sig. (2-tailed)	.454		.013	.850	.440	.454	.125
	N	18	18	18	18	18	18	18
Chla	Pearson Correlation	-.177	.571*	1	-.217	-.098	.107	.501*
	Sig. (2-tailed)	.482	.013		.387	.699	.671	.034
	N	18	18	18	18	18	18	18
Carotenoids	Pearson Correlation	.418	-.048	-.217	1	.123	.588*	-.555*
	Sig. (2-tailed)	.084	.850	.387		.626	.010	.017
	N	18	18	18	18	18	18	18
TBARS	Pearson Correlation	-.189	-.194	-.098	.123	1	.127	-.057
	Sig. (2-tailed)	.452	.440	.699	.626		.615	.822
	N	18	18	18	18	18	18	18
H2O2	Pearson Correlation	.415	-.188	.107	.588*	.127	1	-.329
	Sig. (2-tailed)	.087	.454	.671	.010	.615		.183
	N	18	18	18	18	18	18	18
Phenols	Pearson Correlation	-.425	.376	.501*	-.555*	-.057	-.329	1
	Sig. (2-tailed)	.079	.125	.034	.017	.822	.183	
	N	18	18	18	18	18	18	18

*. Correlation is significant at the 0.05 level (2-tailed). 16
